# Effects of synthetic colloids on oxidative stress and inflammatory response in hemorrhagic shock: comparison of hydroxyethyl starch 130/0.4, hydroxyethyl starch 200/0.5, and succinylated gelatin

**DOI:** 10.1186/cc12820

**Published:** 2013-07-12

**Authors:** Gan Chen, Guoxing You, Ying Wang, Mingzi Lu, Weina Cheng, Jing Yang, Lian Zhao, Hong Zhou

**Affiliations:** 1 Institute of Transfusion Medicine, Academy of Military Medical Sciences, No. 27th Taiping Road, HaiDian, Beijing, China; 2 Department of Biological Engineering, College of Environment and Chemical Engineering, Yanshan University, No. 438 Hebei Street, Qinhuangdao, Hebei, China

**Keywords:** Hemorrhagic shock, Fluid resuscitation, Multiple organ failure, Hydroxyethyl starch, Oxidative stress, Inflammatory response

## Abstract

**Introduction:**

This study compared the effects of hydroxyethyl starch 130/0.4, hydroxyethyl starch 200/0.5, and succinylated gelatin on oxidative stress and the inflammatory response in a rodent hemorrhagic shock model.

**Methods:**

Sodium pentobarbital-anesthetized adult male Wistar rats (200 g to 220 g) were subjected to a severe volume-controlled hemorrhage using arterial blood withdrawal (30 mL/kg to 33 mL/kg) and resuscitated with a colloid solution at the same volume as blood withdrawal (hydroxyethyl starch 130/0.4, hydroxyethyl starch 200/0.5, or succinylated gelatin). Arterial blood gas parameters were monitored. Malondialdehyde (MDA) content and myeloperoxidase (MPO) activity in the liver, lungs, intestine, and brain were measured two hours after resuscitation. The levels of tumor necrosis factor-alpha (TNF-α) and interleukin-6 in the intestine were also measured.

**Results:**

Infusions of hydroxyethyl starch 130/0.4, but not hydroxyethyl starch 200/0.5 or succinylated gelatin, significantly reduced MDA levels and MPO activity in the liver, intestine, lungs and brain, and it also inhibited the production of TNF-α in the intestine two hours after resuscitation. However, no significant difference between hydroxyethyl starch 200/0.5 and succinylated gelatin was observed.

**Conclusions:**

Hydroxyethyl starch 130/0.4, but not hydroxyethyl starch 200/0.5 or succinylated gelatin, treatment after hemorrhagic shock ameliorated oxidative stress and the inflammatory response in this rat model. No significant differences were observed after hydroxyethyl starch 200/0.5 or succinylated gelatin administration at doses of approximately 33 mL/kg.

## Introduction

Hemorrhagic shock (HS) is the leading cause of death in civilian and military trauma [1,2]. Initial survivors of HS are particularly susceptible to the systemic inflammatory response syndrome (SIRS), which triggers multiple organ failure (MOF) and post-traumatic death [3-5]. The pathogenesis of multiple organ failure remains elusive, but the oxidative stress and systemic inflammation that are induced by hemorrhagic shock/fluid resuscitation (HS/R) contribute to the occurrence of MOF [6,7].

Fluid resuscitation is a common intervention for the management of HS victims to maintain organ perfusion, particularly on the battlefield. However, fluid resuscitation may contribute to oxidative stress and inflammation due to reperfusion injury. Oxidative stress induced by reactive oxygen species (ROS) may directly damage cellular membranes via lipid peroxidation. Oxidative stress also initiates systemic inflammatory cascades through the enhancement of neutrophil activation [8]. Activated neutrophils release cytotoxic ROS, proteases and elastases which produce tissue injury, an enhanced systemic inflammatory response and MOF [9,10]. Therefore, ideal resuscitation strategies should suppress oxidative stress and the systemic inflammatory response after HS in addition to maintaining effective organ perfusion. Colloid solutions are widely used for the prevention and correction of hypovolemia in clinical fluid management. However, direct comparisons of oxidative stress and the inflammatory response after commonly used synthetic colloid infusions in hemorrhagic shock are lacking.

Our understanding of the important role of resuscitation fluids in the pathogenesis of MOF raises questions on the effect of widely used colloid fluids in the suppression of oxidative stress and the inflammatory response in vital tissues. We hypothesized that oxidative stress and the inflammatory response is influenced by the choice of colloid solutions. Hydroxyethyl starch (HES), gelatin (GEL), and dextrans are commonly used synthetic colloids [11,12]. HES 130, HES 200, succinylated GEL, and dextran 70 are four representative products. The present study in a rat HS model compared HES 130, HES 200, and GEL to demonstrate their relative therapeutic benefits in the amelioration of HS/R-induced oxidative stress and the inflammatory response. Dextran 70 was not included in this experiment because it is not tolerated by rats [13,14]. Malondialdehyde (MDA), which is a product of lipid peroxidation, was measured to determine the oxidative stress in tissues in this study. Myeloperoxidase (MPO) activity was measured to determine neutrophil sequestration in tissues [15]. The levels of tumor necrosis factor alpha (TNF-α) and interleukin-6 (IL-6) in the intestine were also measured.

## Materials and methods

### Animals

The ethics committee of the Institute of Transfusion Medicine, Academy of Military Medical Sciences approved the study methods. All efforts were made to minimize the number of animals used and their suffering. Thirty-one male Wistar rats weighing 200 g to 220 g (Vital River Laboratories, Beijing, China) were used after a minimum five- to seven-day acclimation period at 25°C in a 12-hour light/dark cycle.

### Surgical procedures

The rats were anesthetized with intraperitoneal injections of sodium pentobarbital (50 mg/kg) and allowed to breathe spontaneously in a supine position on a heating pad (SOFTRON, TMS-201, Beijing, China) that was maintained at 37°C ± 0.1°C throughout the study. The right femoral artery and vein were catheterized with polyethylene catheters (PE-50). Supplementary doses of pentobarbital were administered when necessary.

### Hemorrhagic shock protocol

**Figure 1:**
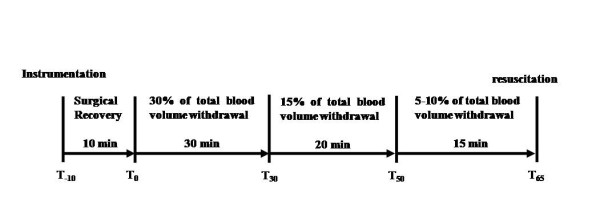
**Design of the hemorrhagic shock protocol**.

A rat HS model was prepared as described previously [16] with modifications. A volume-controlled hemorrhage of 18 mL/kg (approximately 30% of total blood volume) was performed for 30 minutes through the right femoral arterial catheter after surgical preparation and 10 minutes of stabilization. The animals were subjected to a slower hemorrhage of 12 mL/kg to 15 mL/kg (approximately 20% to 25% of total blood volume) for 35 minutes (Figure 1). Hemorrhage was performed using pumps (Softron Beijing, Inc., Beijing, China). The rats with a base excess (BE) of -9 mmol/L to -12 mmol/L were resuscitated via the femoral vein after blood withdrawal.

### Resuscitation groups

The animals were randomized into the following four groups: 1) sham group that underwent all instrumentation procedures without blood collection (*n *= 7); 2) HES 130 group, volume resuscitation with 6% HES 130/0.4 (Voluven^®^, Fresenius-Kabi, Bad Homberg, Germany) (*n *= 8); 3) HES 200 group, volume resuscitation with 6% HES 200/0.5 (HAES-steril^®^, Fresenius-Kabi, Bad Homberg, Germany) (*n *= 8); and 4) GEL group, resuscitation with 4% succinylated GEL (Gelofusine^®^, B. Braun, Shenyang, China) (*n *= 8). The rats were resuscitated using the same volume of synthetic colloid solutions as the volume of blood withdrawn. **A**ll infusions were performed using a pump driven at a constant rate of 0.33 mL/minute over 20 minutes in all groups.

### Blood and tissue sampling

Blood gas analysis was performed at baseline, after blood withdrawal and two hours after resuscitation using 0.25 mL of arterial blood with a blood gas analyzer (ABL80 FLEX, Radiometer Copenhagen, Denmark). All of the animals were euthanized by exsanguination under anesthesia two hours after resuscitation. Tissue samples were washed with cold saline, snap-frozen in liquid nitrogen, and stored in liquid nitrogen until assayed.

### Measurement of MDA and MPO activity levels

Tissues were homogenized and sonicated on ice in 0.9% saline. The homogenates were centrifuged at 1,500 g for 15 minutes at 4°C. The supernatants were used for the measurement of MDA levels and MPO activity (Jiancheng Biological Institute, Nanjing, China) using colorimetric determination according to the manufacturer's recommendations.

### Measurement of inflammatory cytokines levels

The intestinal levels of TNF-α and IL-6 were determined using an enzyme-linked immunosorbent assay (ELISA) kit (PeproTech, Rocky Hill, NJ, USA) according to the manufacturer's instructions. Briefly, the intestine was homogenized on ice in 0.9% saline containing a protease inhibitor cocktail (Roche, Mannheim, Germany). The homogenates were centrifuged at 1,500 g for 15 minutes at 4°C, and the supernatants were assayed for TNF-α and IL-6. Values are expressed as pg/mg protein.

### Statistical analysis

Results are expressed as the means±SD. All data were examined for normal distribution and homogeneity of variance and analyzed using analysis of variance with *post hoc *least significant difference when normality and homogeneity of variance assumptions were satisfied; otherwise, the non-parametric Kruskal-Wallis test was applied. Blood gas variables were studied using the variance analysis test for repeated measurements. *P *values <0.05 were considered significant. Data were analyzed using SPSS Version 18 (SPSS, Chicago, IL, USA).

## Results

### Blood gas analysis

The pH, pCO_2_, pO_2 _and BE values were not different between groups at baseline (Table 1). No significant differences in pH, pCO_2_, pO_2 _and BE values were observed in the groups that underwent hemorrhagic shock at the end of hemorrhage. pH, pCO_2_, and BE values and hemoglobin content decreased significantly at the end of hemorrhage in hemorrhagic groups, but the pO_2 _value increased in these groups (*P *<0.05 versus baseline). Resuscitation increased pH values (*P *<0.05 versus baseline and after hemorrhage). Moreover, the pH value was higher in the HES 130 group than in the HES 200 and sham groups (*P *<0.05). Infusion of HES 130 and GEL decreased the pO_2 _(*P *<0.05 versus after hemorrhage), but the pO_2 _values were not different among the HS/R groups. BE values improved at the end of the experiment in the HES 130, HES 200, and GEL groups (*P *<0.05 versus after hemorrhage). However, no pronounced differences were observed among the three groups. Hemoglobin content exhibited a further decrease when the animals were resuscitated with fluids (*P *<0.05 versus after hemorrhage).

**Table 1:** Arterial blood gas variables for all groups during the experiment

Variables	Groups	Baseline	After hemorrhage	Two hours post-resuscitation
pH	SHAM	7.41 ± 0.02	-	7.44 ± 0.03^a^
	GEL	7.42 ± 0.02	7.36 ± 0.05^a^	7.48 ± 0.04^ab^
	HES 200	7.42 ± 0.03	7.34 ± 0.05^a^	7.46 ± 0.02^ab^
	HES 130	7.42 ± 0.03	7.35 ± 0.03^a^	7.4 9 ± 0.03^abcd^
pO_2 _(Torr)	SHAM	74.9 ± 5.3	-	81.0 ± 9.5
	GEL	78.5 ± 4.5	109 ± 3.9^a^	87.7 ± 11.8^b^
	HES 200	78.5 ±8.5	105.2 ± 12.8^a^	91.1 ± 18.8
	HES 130	79.0 ± 6.5	106.5 ± 13.7^a^	91.0 ± 5.5^abc^
pCO_2 _(Torr)	SHAM	39.0 ± 3.1	-	36.7 ± 3.7
	GEL	39.8 ± 3.6	25.3 ± 3.8^a^	29.3 ± 4.0^ac^
	HES 200	39.6 ± 3.6	28.9 ± 3.2^a^	30.7 ± 2.7^ac^
	HES 130	40.2 ± 3.0	28.3 ± 4.4^a^	28.0 ± 2.7^ac^
Hemoglobin (g/dl)	SHAM	12.4 ± 0.7	-	12.6 ± 0.5
	GEL	12.9 ± 1.3	10.0 ± 0.6^a^	6.8 ± 0.7^abc^
	HES 200	13 ± 0.4	9.7 ± 1.0^a^	6.7 ± 0.7^abc^
	HES 130	13.4 ± 0.5	9.8 ± 0.5^a^	7.2 ± 0.7^abc^
BE (mmol/L)	SHAM	0.4 ± 2	-	1.3 ± 1.8
	GEL	1.5 ± 1.9	-9.9 ± 2.1^a^	-1.6 ± 2.1^abc^
	HES 200	1.5 ± 1.0	-9.3 ± 2.39^a^	-1.7 ± 1.8^abc^
	HES 130	1.8 ± 1.6	-9.2 ± 2.09^a^	-1.6 ± 2.4^abc^

^a^*P *<0.05 versus baseline, ^b^*P *<0.05 versus after hemorrhage, ^c^*P *<0.05 versus the sham group, ^d^*P *<0.05 versus the HES 200 group. Values are means ± SD. BE, base excess; pCO_2_, partial pressure of carbon dioxide; pO_2_, partial pressure of oxygen.

### Tissue lipid peroxidation levels

**Figure 2:**
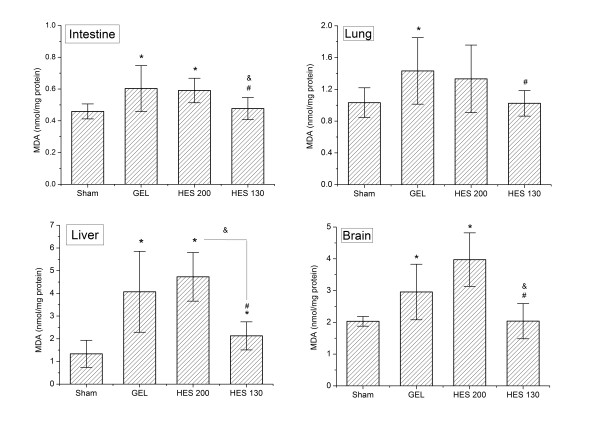
**Tissue MDA concentrations following HS/R**. Data are plotted as the means ± SD. *P <0.05 versus the sham group; &*P *<0.05 versus the HES 200 group; #*P *<0.01 versus the GEL group. GEL, gelatin; HES, hydroxyethyl starch; HS/R, hemorrhagic shock/fluid resuscitation; MDA, malondialdehyde.

MDA concentrations in the liver, lungs, intestine and brain of rats that were resuscitated with HES 130 were all significantly lower compared to the GEL group (*P *<0.05; Figure 2). HES 130 significantly suppressed the elevation of MDA levels in the liver, intestine, and brain compared to HES 200 (*P *<0.01), but similar MDA levels were observed in the lungs. No significant differences were observed between the HES 200 and GEL groups in all tissues.

### Tissue neutrophil accumulation

**Figure 3:**
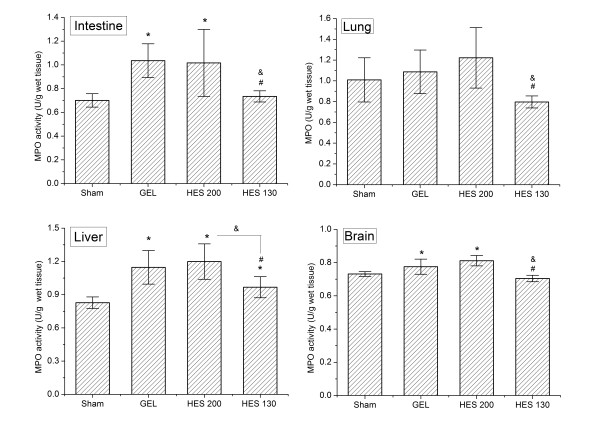
**MPO levels in the liver, lungs, intestine and brain following HS/R in four groups**. Data are plotted as the means ± SD. *P <0.05 versus the sham group; &*P *<0.05 versus the HES 200 group; #*P *<0.01 versus the GEL group. GEL, gelatin; HES, hydroxyethyl starch; HS/R, hemorrhagic shock/fluid resuscitation; MPO, myeloperoxidase.

MPO activity in the liver, lungs, intestine, and brain in the HES 130 group was significantly reduced compared to the HES 200 group (*P *<0.05; Figure 3). The infusion of HES 130 also decreased MPO activity in all measured tissues compared to the GEL group (*P *<0.05). No significant difference between the HES 200 and GEL groups were observed in all four tissues.

### Intestinal levels of inflammatory cytokines

**Figure 4:**
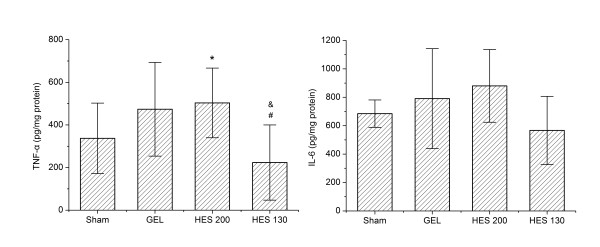
**Effects of different resuscitation fluids on HS/R-induced intestinal levels of TNF-**α **and IL-6**. Data are plotted as the means ± SD. *P <0.05 versus the sham group; &*P *<0.05 versus the HES 200 group; #*P *<0.01 versus the GEL group. GEL, gelatin; HES, hydroxyethyl starch; HS/R, hemorrhagic shock/fluid resuscitation.

The intestinal TNF-α elevation was significantly suppressed in the HES 130 group compared to the HES 200 group (*P *<0.05; Figure 4). Intestinal TNF-α was also lower in the HES 130 group than in the GEL group (*P *<0.05). However, no statistically significant differences in the TNF-α level were observed between the HES 200 and GEL groups. The HES 130 group show a trend for decrease in the IL-6 level compared to the HES 200 and GEL groups, but there was no statistically significant difference.

## Discussion

The present study demonstrated that HES 130 infusion suppressed oxidative stress and the inflammatory response in a rodent model of controlled hemorrhage compared to HES 200 and GEL. No significant differences were observed between HES 200 and GEL.

Prolonged organ ischemia due to hemorrhagic shock may lead to death. Therefore, early aggressive fluid resuscitation for adequate tissue and cellular perfusion has been the therapeutic norm in hemorrhagic shock patients. However, this notion has been challenged recently [17]. Laboratory efforts directed toward the discovery of the ideal resuscitative fluid have emerged from an understanding of hemorrhagic shock as a disease of decreased perfusion and altered immunity. Therefore, research efforts aimed at the identification of treatments for hemorrhagic shock have targeted volume restoration and the prevention and amelioration of the immune and inflammatory effects of hemorrhage [18]. Crystalloids differentially influence hemorrhage-induced oxidative stress and inflammatory responses through the upregulation of ROS generation and neutrophil activity [10,19].

HES solutions are synthetic colloids that are widely used to maintain or improve tissue perfusion in HS treatment. However, the pharmacology of HES varies greatly between solutions depending on their characteristics, including molecular weight, the degree of hydroxyethyl substitution and the C2/C6 ratio of hydroxyethylation [20]. HES 200 and HES 130 solutions are commonly used HES solutions. Blood loss and transfusion requirements are significantly reduced in major surgery when HES 130 is administered compared to HES 200 [21,22]. Furthermore, platelet dysfunction exhibits a faster recovery after the infusion of HES 130 compared to HES 200 [23]. However, little is known about the effects of the two different HES solutions on oxidative stress and the inflammatory response following HS/R.

HES 200 decreases the plasma levels of coagulation factor VIII and von Willebrand factor; this produces coagulation impairment [21,24] and bleeding events [25] compared to HES 130. Extensive cross-talk is observed between inflammation and hemostasis [26]. Vancine *et al*. demonstrated that factor VIII deficiency is associated with higher inflammatory levels after a lipopolysaccharide (LPS) challenge [27]. This result suggests that the impairment in the blood coagulation system results in a relatively poor anti-oxidative and anti-inflammatory effect following HS/R for HES 200. Moreover, the advantages of HES 130 may be due to improvements in tissue oxygen [28] due to its hemorheological advantages over HES 200 [29]. Our results partially confirmed a previous study. Huter *et al*. demonstrated that 6% HES 130/0.42, which is another rapidly degradable HES solution with low molecular weight and degree of substitution, significantly reduces macrophage infiltration and interstitial cell proliferation compared to 10% HES 200/0.5 in an isolated kidney perfusion model [30].

HES 200 exhibits anti-inflammatory effects *in vivo *and *in vitro *[31-34]. In an HS model, a 20 mL/kg HES 200 infusion inhibits the inflammatory response following HS/R compared to GEL [17]. Tsai *et al*. demonstrated that resuscitation using 8 mL/kg HES 200 prevents oxidative stress and nuclear factor-kappa B (NF-κB) activation [35]. HES 200 attenuates cell injury in inflammatory-stimulated tubular epithelial cells *in vitro *[31]. However, these results are not consistent with our observations. We did not observe any differences in oxidative stress and inflammatory responses between the GEL and HES 200 groups. The infusion dose may account for these controversial results. Approximately 33 mL/kg of the colloid solutions were used in this study, which is the recommended maximum dose for HES 200, but it is far less than the recommended maximum daily dose of HES 130 (50 mL/kg) [36]. Tian *et al*. demonstrated that a lower dose of HES 200 (3.75 and 7.5 mL/kg) significantly suppresses LPS-induced NF-κB activation in four tissues (lungs, liver, heart, and kidneys). However, 15 mL/kg HES 200 inhibits NF-κB activity only in the lungs and liver, and 30 mL/kg HES 200 exerts no effect in any measured organs [35,37]. The effect of GEL on the inflammatory response was similar at three doses (7.5, 15 and 30 mL/kg) [38]. These results indicate that the anti-inflammatory effect of HES 200 is volume-dose dependent. A large infusion dose may inhibit the anti-inflammatory effects of HES 200, which produces similar oxidative stress and inflammatory responses between the GEL and HES 200 groups. Future studies are required to delineate the underlying mechanism.

The comparison of the impact of HES 130 and GEL on oxidative stress and the inflammatory response in hemorrhagic shock demonstrated conclusive protective effects after the HES 130 infusion. This result is consistent with previous studies in other models [38,39] and clinical cardiac surgery [40]. HES 130 inhibits the inflammatory response and NF-κB activation in a rat model of polymicrobial sepsis but GEL does not [38]. Varga *et al*. demonstrated that HES 130 prevents ischemia reperfusion-induced leukocyte reactions compared to GEL [39]. One or more protective mechanisms may come into play. Firstly, the animal peptide nature of GEL may render an enhanced immunogenicity compared to HES 130 [17]. Secondly, HES 130, but not GEL, exerts its protective effects via a direct interaction with the vascular endothelium and attenuates leukocyte-endothelial cell interactions [39]. HES 130 dampens HS/R-induced acute neutrophil tissue accumulation [15,40].

The gut is a critical organ that is damaged by HS/R. The postischemic intestine releases proinflammatory molecules, such as superoxide radicals and cytokines, into the portal and systemic circulation, which produces gut-induced remote organ failure [41]. Previous reports have suggested a strong association between intestinal-reperfusion injury and acute damage to the lungs or liver [42-44]. An infusion of HES 130 in this study inhibited the release of proinflammatory cytokines, such as TNF-α and IL-6, which are responsible for gut barrier dysfunction after HS/R [45,46]. This result suggests a protective effect on gut barrier integrity, which reduced the levels of oxidative stress and inflammatory response in the liver, lungs and brain, and the occurrence of MOF. Additional studies on the long-term effects of resuscitation fluids should clarify whether HES 130 can prevent MOF.

Despite the Advanced Trauma Life Support course recommendations of lactated Ringer's solution, a strong case can be made for the use of colloids for initial resuscitation in austere settings, such as battlefield care, in which volume is necessarily limited. Actually, some colloid solutions have been recommended in the resuscitation of hemorrhagic shock in Tactical Combat Casualty Care [18,47,48].

The severity of HS was determined by bled volume and BE in this experiment. BE is an expedient and sensitive measure of both the degree and duration of hypoperfusion [49-51]. It could be a useful guide to volume replacement in the resuscitation of trauma patients and endpoints of resuscitation [52-54].

Our study has some limitations. The comparison results were obtained based on a rat volume-controlled model, which is modified to be more representative of traumatic hemorrhage, and need to be verified in a clinical study. Moreover, the maximal inflammatory and oxidative reaction seems to occur within two hours post-resuscitation in most studies [55,56]. The present study examined only a single time point, that is, two hours after treatment. Thus, further studies about the long-term effects of these colloid solutions, especially the impact on organ function, are needed.

## Conclusions

The current experimental data indicate that resuscitation after hemorrhagic shock with HES 130 attenuated oxidative stress and the inflammatory response in tissues following HS/R compared to HES 200 and GEL. No significant differences in oxidative stress and the inflammatory response were observed after 33 mL/kg HES 200 and GEL infusions. However, the efficacy of these colloids must be proved in the clinical arena. Therefore, further randomized trials are required.

## Key messages

• Infusions of HES 130/0.4, but not 200/0.5 or GEL, significantly reduced MDA levels and MPO activity in the liver, intestine, lungs and brain.

• Infusions of HES 130/0.4, but not HES 200/0.5 or GEL, significantly inhibited the production of TNF-α in the intestine two hours after resuscitation.

• No significant differences were observed after HES 200/0.5 or GEL administration at doses of approximately 33 mL/kg in a rat volume-controlled model.

## Abbreviations

BE: base excess; GEL: gelatin; HES: hydroxyethyl starch; HS: hemorrhagic shock; HS/R: hemorrhagic shock/fluid resuscitation; IL-6: interleukin-6; LPS: lipopolysaccharide; MDA: malondialdehyde; MOF: multiple organ failure; MPO: myeloperoxidase activity; NF-κB: nuclear factor kappa B; pCO_2: _partial pressure of carbon dioxide; pO_2: _partial pressure of oxygen; ROS: reactive oxygen species; SIRS: systemic inflammatory response syndrome; TNF-α: tumor necrosis factor-alpha.

## Competing interests

The authors declare that they have no competing interests.

## Authors' contributions

GC conceived the study, carried out the studies and wrote the manuscript. GY and ML performed the surgical procedures. YW participated in the revision of the manuscript, helped to draft the manuscript and helped to measure MDA and MPO activity levels. WC participated in the revision of the manuscript, helped to draft the manuscript and helped to measure inflammatory cytokines levels. JY contributed to the study statistical analyses. LZ and HZ are the Principal Investigators and take responsibility for all conceptual and technical aspects of this study. All authors have read and approved the final manuscript.
